# Long-term effectiveness and safety of lacosamide as adjunctive therapy in children and adolescents with refractory epilepsy: a real-world study

**DOI:** 10.1186/s12887-023-04039-5

**Published:** 2023-05-20

**Authors:** Ting Zhao, Lu-hai Yu, Hui-lan Zhang, Jing Yu, Jie Feng, Ting-ting Wang, Yan Sun, Hong-jian Li

**Affiliations:** 1 Department of Pharmacy, People’s Hospital of Xinjiang Uygur Autonomous Region, Urumqi, 830001 Xinjiang China; 2 Institute of Clinical Pharmacy of Xinjiang Uygur Autonomous Region, People’s Hospital of Xinjiang Uygur Autonomous Region, Urumqi, 830001 Xinjiang China; 3 Department of Neurology, Children’s Hospital of Xinjiang Uygur Autonomous Region, Xinjiang Hospital of Beijing Children’s Hospital, Urumqi, 830001 Xinjiang China

**Keywords:** Adolescents, Children, Effectiveness, Lacosamide, Refractory epilepsy

## Abstract

**Purpose:**

The effectiveness and tolerability of lacosamide (LCM) among Chinese children and adolescents with refractory epilepsy has not yet been established. Therefore, the objective of this study was to assess the effectiveness and tolerability of LCM among children and adolescents with refractory epilepsy in Xinjiang, Northwest China.

**Methods:**

Effectiveness was assessed by measuring changes in seizure frequency at 3, 6 and 12 months compared with baseline. Patients that achieved ≥ 50% reduction in the frequency of all seizures per month, relative to baseline, were considered to be responders.

**Results:**

105 children and adolescents with refractory epilepsy were enrolled in the study. The responder rates were 47.6%, 39.2%, and 31.9%, respectively at 3, 6, and 12 months. Seizure freedom rates were 32.4%, 28.9%, and 23.6% at 3, 6, and 12 months, respectively. The retention rates at 3, 6, and 12 months were 92.4%, 78.1%, and 69.5%, respectively. The maintenance dose of LCM within the responder group (8.2 ± 4.5 mg·kg^− 1^·d^− 1^) was significantly higher compared to the non-responder group (7.3 ± 2.3 mg·kg^− 1^·d^− 1^) (p < 0.05). At first follow-up, 44 patients (41.9%) reported experiencing at least one treatment-emergent adverse events.

**Conclusion:**

This real-world study of children and adolescents validated that LCM was both an effective and well-tolerated treatment option for the treatment of refractory epilepsy.

## Introduction

Pediatric patients are more likely to suffer from epilepsy compared to adults, and it has been estimated that approximately 6 million pediatric patients in China suffer from epilepsy, which leads to a prevalence of approximately 7 per 10,000 Chinese pediatric patients [1]. Lacosamide (LCM), (R)-2-acetamido-N-benzyl-3-methoxy- propionamide), is a third-generation antiseizure medication (ASM) approved by the European Medicines Agency and US Food and Drug Administration [2, 3]. In China, LCM has been approved for the treatment of focal-onset seizures, with or without secondary generalization in adults, adolescents, and pediatric patients from 4 years of age. This new indication has greatly accelerated the use of LCM in children and adolescents with epilepsy over 4 years of age, which enables physicians to use more therapies for the treatment of refractory epilepsy.

Clinical studies have indicated a favorable short- and long-term effectiveness, as well as tolerability of lacosamide [4–7]. In regulatory randomized controlled trials conducted in adults, lacosamide has demonstrated to be an effective and safe ASM, with 40% of patients with refractory focal epilepsy achieving a 50% reduction in seizure frequency at a short period of time (3 months) [8]. The evidence of lacosamide effectiveness in Chinese paediatric patients with refractory epilepsy is scarce. Therefore, some pediatric clinicians have questioned whether LCM can be used as an add-on treatment for refractory epilepsy in children and adolescents over 4 years of age. As LCM was previously approved in China in 2018, and then approved for combined treatment of focal seizures among epilepsy patients aged 4 years and older, publishing real world experience can support the wider use of the product as it allows prescribers to learn about its profile.

Thus far, there has been limited data on the use of LCM among Chinese pediatric patients with refractory epilepsy. Therefore, the objective of this study was to assess the effectiveness and tolerability of LCM for the first time among children and adolescents with refractory epilepsy in Xinjiang, Northwest China.

## Materials and methods

### Collection of demographic details of the patients

This was a retrospective, observational study among children and adolescents with refractory epilepsy, conducted under normal clinical practice at two hospitals in Xinjiang, China. All children and adolescents met the diagnostic criteria for epilepsy, as issued by the International League against Epilepsy in 2017 [9].

The inclusion criteria were as follows: treatment with LCM for refractory epilepsy, and treatment with LCM for at least 2 weeks. The exclusion criteria were as follows: the lack of key research data, cognitive impairment, alcohol or drug abuse within the last 5 years, if patients had any medical or psychiatric condition.

The International League Against Epilepsy (ILAE) defines refractory epilepsy as: after a reasonable selection according to the type of epilepsy and correct use of at least 2 well-tolerated ASMs (single or combined), the patient’s seizure-free duration did not reach three times the longest seizure interval before treatment or one year (depending on whichever was longer) [9].

Anonymized information was retrospectively gathered from medical records without the involvement or participation of any other individuals, with a study cutoff date of May 2022. All methods were performed in accordance with the relevant guidelines and regulations (Declaration of Helsinki). This study was approved by the Ethics Committee of People’s Hospital of Xinjiang Uygur Autonomous Region (Xinjiang, China; Ethical Approval number: KY2019120614).

The need for informed consent was waived by the ethics committee/Institutional Review Board of People’s Hospital of Xinjiang Uygur Autonomous Region, because of the retrospective nature of the study.

Data collected included age, sex, height, weight, body mass index (BMI), age at seizure onset and duration of epilepsy, final LCM dosage and adverse events that occurred at any time during the treatment.

### Effectiveness

Seizure frequency was recorded at an average per month for the past 3 months at baseline, and at each follow-up period for 3, 6, and 12 months. Effectiveness was assessed by measuring changes in seizure frequency at 3, 6 and 12 months’ follow up compared with baseline. The baseline was 3 months before the addition of LCM and the seizure frequency was based on the patients’ seizure diary. Patients that achieved ≥ 50% reduction in the frequency of all seizures per month, relative to baseline, were considered to be responders. The term “seizure-free” was defined as having complete seizure control using LCM.

Seizure frequency was assessed through documented ‘seizure diary’ and subjects or caregiver reports. Seizure frequency was classified as monthly (1–3 seizures per month) or yearly (≤ 12 seizures per year). To avoid possible influence on treatment decisions, the last documented visit had to be performed prior to initiation of the chart review. Most of the clinical records at our study did not document seizure frequency in a standardized way, such as “there was a decrease in seizures”, “no seizures since last visit”, and “four seizures since last visit”. These narrative descriptions are very inconsistent. Therefore, in addition to inquiring clinical records, we also inquired and recorded the frequency of epileptic seizures through telephone follow-up.

### Safety assessments

The safety and tolerability depended on type and frequency of any one adverse event during epilepsy treatment across all patients, as well as LCM-related events that were recorded at any time from start of LCM treatment to 12 months adverse events [10]. Adverse reactions that led to LCM discontinuation were also noted. The source of the data to assess safety were “seizure diaries” and “clinical inpatient/outpatient records” recorded by families of children with epilepsy. These data specifically recorded adverse events in people with epilepsy and were categorized at the time of retrospectively. Considering safety assessment, our approach to ruling out other possible causes was whether symptoms disappearing after LCM withdrawal.

All psychiatric adverse reactions in pediatric patients were diagnosed by clinical psychologists according to the “Chinese Expert Consensus on the Diagnosis and Treatment of Epilepsy and Depression (2022 Revised Edition)”, and “Canadian Network for Mood and Anxiety Treatments (CANMAT) and International Society for Bipolar Disorders (ISBD) 2018 guidelines for the management of patients with bipolar disorder”. Psychiatric adverse events in the treatment of LCM mainly refer to depression and bipolar disorder.

### Statistical analysis

Analyses were performed using SPSS version 19.0 software (version 4.0.100.1124, Chicago, IL, USA), and a *p*-value of < 0.05 was considered statistically significant. Descriptive statistics for clinical data presentation were applied. Comparisons between groups and response outcomes were made using the chi-square (χ^2^) test or Fisher’s exact test for qualitative variables and Student’s *t*-test or the Mann-Whitney U test for quantitative variables whose distribution was normal and nonnormal, respectively.

## Results

### Study population and baseline characteristics

Overall, from September 2019 to March 2022, 105 children and adolescents from two hospitals were enrolled in the study, demographics and clinical data are summarized in Table 1. The mean age at LCM treatment was 7.6 years (median: 4.5 years), and 64 patients (61.0%) were male. The mean body mass index (BMI) was 18.1 kg·m^− 2^ (median: 17.8). The mean epilepsy duration at LCM treatment was 3.4 years (median: 3.0 years). Median follow-up length from LCM initiation to last evaluation was 0.6 years. Thirty-two patients (30.5%) received LCM as add-on for generalized epilepsy and 12 (11.4%) for focal epilepsy, while 43 patients (41.0%) manifested both generalized and focal seizures (combined epilepsy).

The 105 patients were divided into two groups according to whether the treatment was effective or not. This included the responder group (n = 50), and the non-responder group (n = 55). The results of the Student’s *t*-test and chi-square (χ^2^) test showed no statistically significant significance between responder and non-responder groups for age, body mass index, duration of seizures, duration of seizures, duration of seizures, concomitant ASM, and maintenance LCM (p > 0.05).

However, the chi-square (χ^2^) test showed a significant difference in gender and the number of ASMs were used before LCM between the response group and the non-responder group, the proportion of male patients within the responder group (68.0%) was significantly higher compared to the non-responder group (54.5%) (p = 0.049), the proportion of one ASM used before LCM within the responder group (54.0%) was significantly higher compared to the non-responder group (29.1%) (p < 0.001), and the proportion of two ASMs used before LCM within the responder group (30.0%) was significantly lower compared to the non-responder group (45.5%) (p = 0.023) (Table 1). In addition, the Student’s *t*-test results showed that the maintenance dose of LCM within the responder group (8.2 ± 4.5 mg·kg^− 1^·d^− 1^) was significantly higher compared to the non-responder group (7.3 ± 2.3 mg·kg^− 1^·d^− 1^) (p = 0.019) (Table 1).

**Table 1:** Patient demographics and characteristics, as well as differences between responder group and non-responder group (mean ± standard deviations)

Category	Total population (n = 105)	Responder group (n = 50)	Non-responder group (n = 55)	t /χ^2^	p-value
Age (years)	7.6 ± 4.5	7.3 ± 4.7	7.8 ± 4.5	-0.582	0.562
Male gender, n (%)	64 (61.0)	34 (68.0)	30 (54.5)	3.882	< 0.05*
Body mass index (kg·m^− 2^)	18.1 ± 4.1	17.8 ± 3.4	18.3 ± 4.6	-0.657	0.513
Duration of epilepsy (years)	3.4 ± 2.8	3.1 ± 2.5	3.6 ± 2.7	-1.078	0.284
Medication time (years)	0.6 ± 0.5	0.6 ± 0.4	0.7 ± 0.5	-0.878	0.382
Type of seizure, n (%)					
Generalized onset	32 (30.5)	15 (30.0)	17 (30.9)	0.024	0.877
Focal onset	12 (11.4)	6 (12.0)	6 (10.9)	0.061	0.805
Combined generalized and focal onset	43 (41.0)	21 (42.0)	22 (40.0)	0.083	0.773
Unknow onset	18 (17.1)	8 (16.0)	10 (18.2)	0.142	0.707
Maintenance dose (mg·kg^− 1^·d^− 1^)	7.8 ± 2.4	8.4 ± 2.4	7.3 ± 2.3	2.375	< 0.05*
Concomitant ASMs ^#^					
Valproic acid	92 (38.3)	47 (41.2)	45 (35.7)	0.528	0.467
Levetiracetam	62 (25.8)	32 (28.1)	30 (23.8)	0.416	0.519
Oxcarbazepine	51 (21.3)	20 (17.5)	31 (24.6)	1.540	0.215
Lamotrigine	21 (8.7)	10 (8.8)	11 (8.7)	-	1.000
Perampanel	6 (2.5)	2 (1.7)	2 (1.6)	-	1.000
Zonisamide	5 (2.1)	2 (1.7)	3 (2.4)	-	1.000
Phenobarbital	2 (0.8)	0 (0)	2 (1.6)	2.020	0.155
Topiramate	2 (0.8)	0 (0)	2 (1.6)	2.020	0.155
Clonazepam	1 (0.4)	1 (0.9)	0 (0)	1.005	0.316
The number of ASMs were used before LCM, n (%)					
One ASMs	43 (41.0)	27 (54.0)	16 (29.1)	12.872	< 0.001**
Two ASMs	40 (38.1)	15 (30.0)	25 (45.5)	5.164	< 0.05*
Three ASMs	18 (17.1)	7 (14.0)	11 (20.0)	1.276	0.259
Four ASMs	4 (3.8)	1 (2.0)	3 (5.4)	1.332	0.248

^#^ One patient may have two or more ASMs

Comparison between effective cases, * p-value < 0.05; ** p-value < 0.001

### Retention rates

**Fig. 1:**
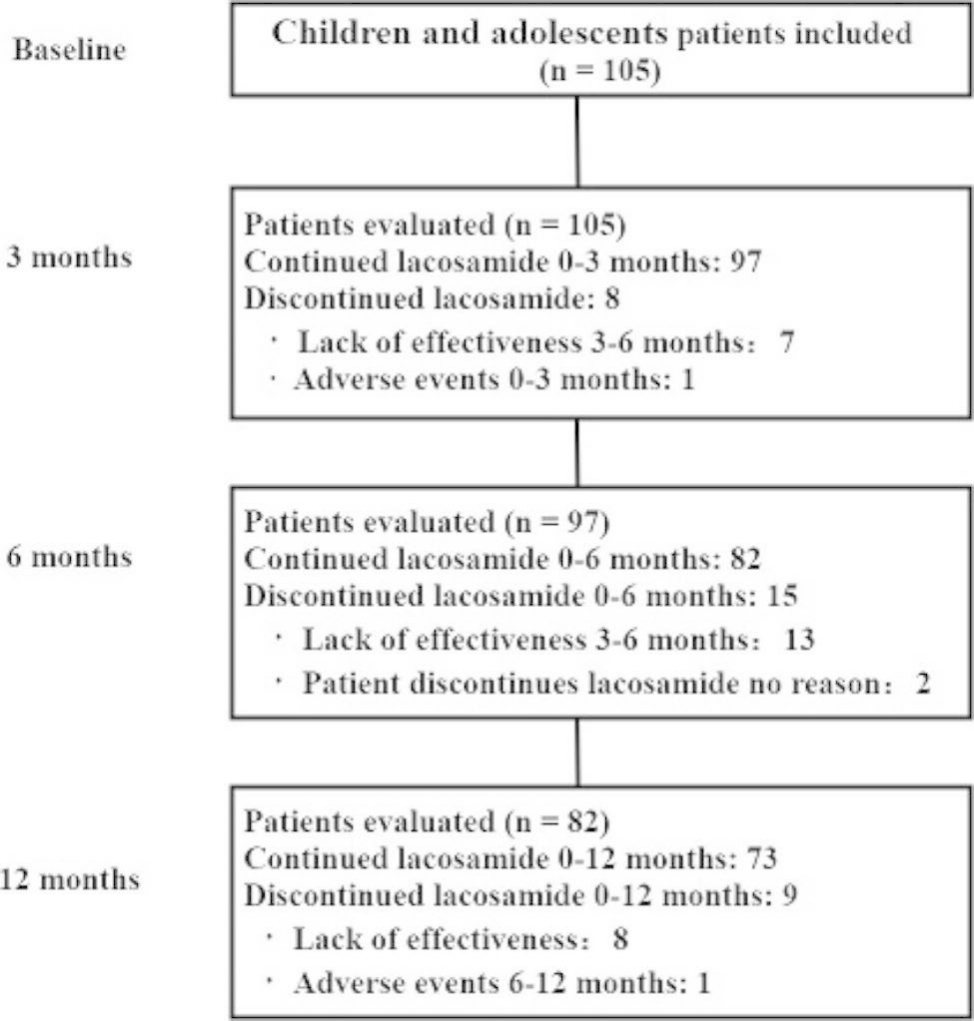
Illustration of the number of patients evaluated at each visit who have been treated with lacosamide treatment at some point during the 3 months and at the 12 months

The median length of LCM treatment administration was 7 months. A follow-up of at least 3 months was available in 105 children and adolescents. The mean maintenance dose of LCM was 8.2 mg·kg^− 1^·d^− 1^ (standard deviation: 2.4). At follow-up within 12 months, 23 patients discontinued LCM treatment (Fig. 1). The retention rates at 3, 6, and 12 months were 92.4%, 78.1%, and 69.5%, respectively.

### Effectiveness and tolerability

**Fig. 2:**
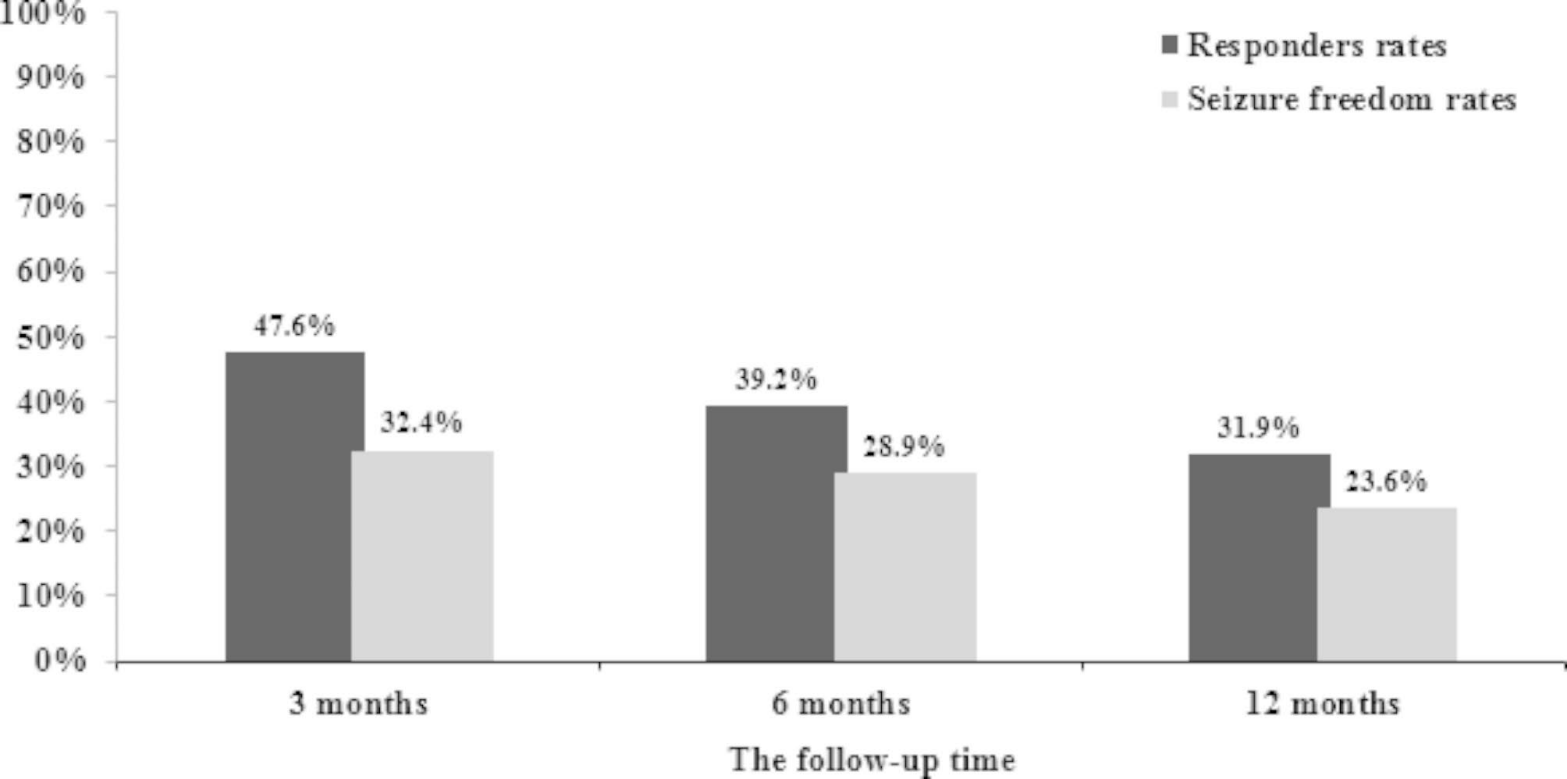
Effectiveness outcome on lacosamide treatment for refractory epilepsy (the total number of people in each group was 105, 97, and 82 patients; the number of responder to lacosamide in each group was 50, 38, and 23 patients; the number of seizure-free to lacosamide in each group was 34, 28, and 17 patients)

The effectiveness of LCM therapy was evaluated in 105 children and adolescents that were enrolled in the study. Among children and adolescents that developed seizures during the baseline period, responder rates for all seizure types at 3, 6, and 12 months were 47.6%, 39.2%, and 31.9%, respectively (Fig. 2). Seizure freedom rates were 32.4%, 28.9%, and 23.6% at 3, 6, and 12 months, respectively (Fig. 2). Overall, 73 patients continued treatment with LCM for 12 months or longer.

**Fig. 3:**
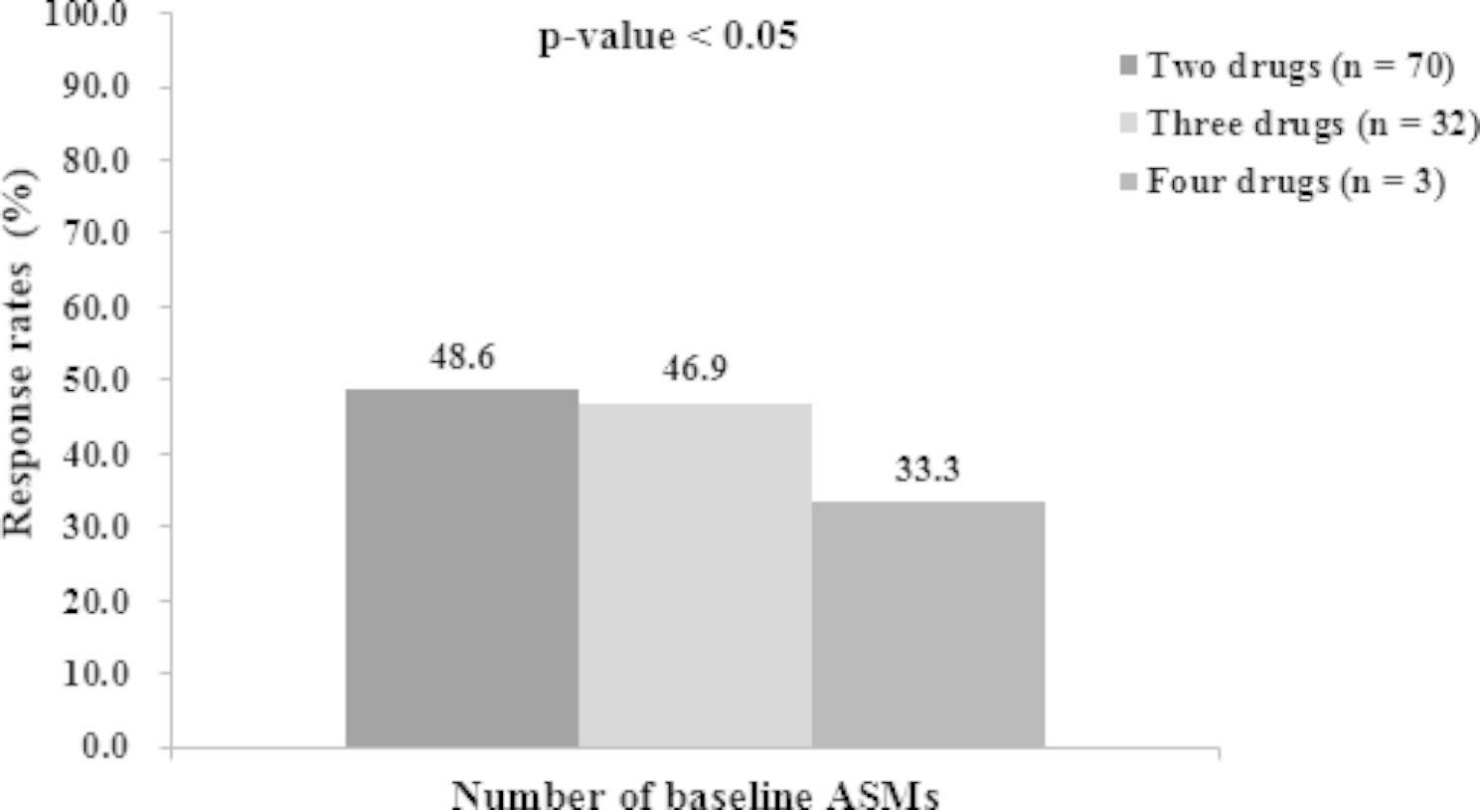
Percentages of children achieving seizure freedom during the last 12-month period stratified by number of baseline ASMs (the total number of people in each group was 70, 32, and 3 patients; the number of seizure-free to lacosamide in each group was 34, 15, and 1 patients)

**Fig. 4:**
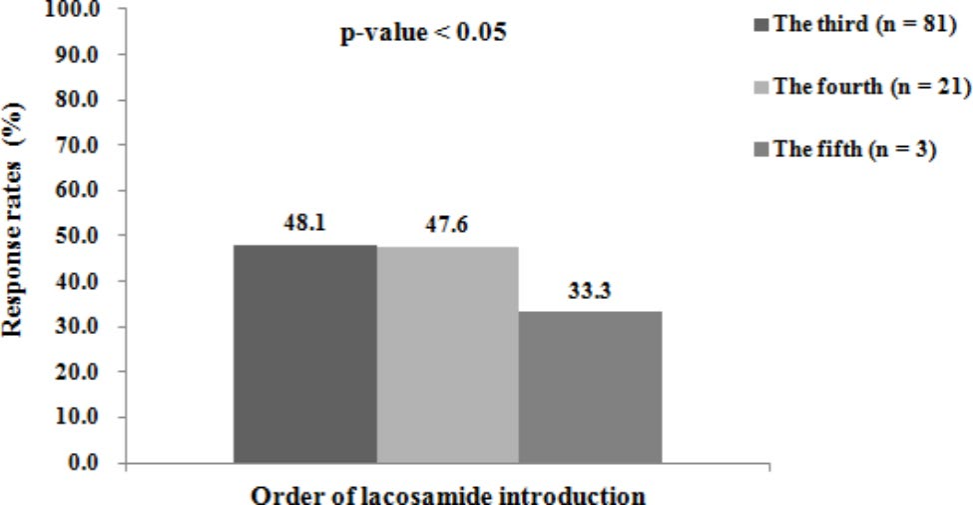
Percentages of children achieving seizure freedom during the last 12-month period stratified according to the order of LCM introduction (the total number of people in each group was 81, 21, and 3 patients; the number of seizure-free to lacosamide in each group was 39, 10, and 1 patients)

Several variables that could potentially affect the likelihood of achieving a seizure remission during the 12-month period were analysed. Patients maintained on a three and four number of baseline ASMs at the time of LCM introduction were more likely to achieve seizure remission (p < 0.05, Fig. 3). The order of LCM introduction was a highly significant factor impacting the likelihood of seizure remission during the 12-month period (p < 0.05, Fig. 4). The earlier LCM was introduced, the higher the likelihood of achieving a remission.

### Safety and tolerability

Clinical data on adverse events are summarized in Table 2. At first follow-up, 44 patients (41.9%) reported experiencing at least one treatment-emergent adverse events, with a mean daily dose of 7.8 mg·kg^− 1^·d^− 1^ (range: 2.8–14.8, standard deviation: 2.5). In most cases, these cases were rated mild. At the 12 months, 28 patients (40.6%) reported experiencing treatment-emergent adverse events at a mean daily dose of 8.3 mg·kg^− 1^·d^− 1^ (range: 3.2–10.7; standard deviation: 2.5), which, in most cases, were rated mild (Table 2).

**Table 2:** Characteristics of adverse drug reactions related to lacosamide at the first follow-up (n = 105) and last follow-up (n = 82)

Adverse drug reactions	At the first follow-up (n, %)	At the 12-months follow-up (n, %)
Any TEAEs	44 (41.9)	28 (34.2)
Most frequent TEAEs (≥ 5% of patients)		
Dizziness	24 (22.9)	18 (18.3)
Somnolence	24 (22.9)	17 (20.7)
Nausea/vomiting	6 (5.7)	3 (3.7)
TEAEs leading to dose adjustment		
Dizziness	2 (1.9)	2 (2.4)
Nausea/vomiting	2 (1.9)	1 (1.2)
Psychiatric	1 (0.9)	1 (1.2)
Double vision	1 (0.9)	0 (0)

TEAEs, treatment-emergent adverse events

During the entire period of LCM treatment, there were no death or severe adverse events reported. Four patients (3.8%) experienced psychiatric adverse events, with one patient presenting with depressive symptoms and three patients with bipolar disorder. Among the nonpsychiatric adverse events, mild dizziness, somnolence, nausea/vomiting and depression were the most common, and were reported by 24, 24, 6 and 4 patients, respectively. Other adverse events included gastrointestinal discomfort in two patients, and double vision in another.

## Discussion

Voltage-gated Na^+^ channels are an important class of therapeutic targets for many anticonvulsant drugs, including both classical anticonvulsants and third-generation ASMs [11]. One such anticonvulsant is LCM, a third-generation ASM that is approved as monotherapy or adjunctive therapy in adults with partial-onset seizures in China. Notably, although many anticonvulsants do not seem to affect Na^+^ channel slow inactivation markedly, LCM shows a pronounced effect on slow inactivation properties [12]. LCM is a functional amino acid that is thought to exert its distinctive anticonvulsant mechanism of action through selective enhancement of slow inactivation of voltage-gated sodium channels [12].

Determining the effectiveness, safety and optimal dosage of LCM among pediatric patients is vital for safe and rational use of LCM in clinical practice. Various endogenous factors (i.e., culture, diet, and health behaviours) and exogenous factors (i.e., race, ethnicity, and environment) can have an effect on drug pharmacokinetics [13]. In recent years, many pivotal, double-blind, placebo-controlled clinical trials have validated the effectiveness and tolerability of LCM among pediatric patients and adolescents with epilepsy [4–7, 14–17]. There are few clinical data on LCM in Chinese pediatric patients with refractory epilepsy. Hence, its safety and effectiveness among Chinese pediatric patients with refractory epilepsy need to be further investigated. This study was conducted during the first two years of introduction of LCM in China, when the majority of physicians were starting to gain experience with this new ASM.

The children and adolescents in this study are representative of the real-world refractory epilepsy population, and despite limited clinical experience with LCM therapy for refractory epilepsy, there is a growing body of research that suggests that LCM may be useful for children and adolescents with refractory epilepsy [8, 21–22]. Our study demonstrates clinical experience that was gained in the first year of LCM therapy for refractory epilepsy.

Rosati et al. discovered that 38.6% (34/88) children and adolescents were responders that received add-on LCM treatment for refractory epilepsy and nine (26.4%) of the 34 responders were seizure-free [18]. Kleist et al. discovered that 48% (38/80) patients with refractory epilepsy were documented as responders after being administered LCM treatment [19]. Meanwhile, Rüegger et al., evaluated the effectiveness of LCM among 107 patients with drug-resistant, and found that 52 (49%) children were continued LCM at last follow-up, and 55 (51%) discontinued LCM during the study period [20].

This real-world study of children and adolescents with refractory epilepsy with a follow-up period of at least 12 months demonstrated response rates of 47.6%, 39.2%, and 31.9% at 3, 6, and 12 months, respectively. In particular, the proportion of pediatric patients who achieved seizure freedom gradually decreased over time, at 32.4%, 28.9%, and 23.6% at 3, 6, and 12 months, respectively. These results were consistent with Rosati *et al.,.* [18] Kleist et al., [19] and Rüegger et al., [20] studies findings. Although there is no definite explanation for these results, they are thought to be related to the development of focal tolerance to the long-term treatment of LCM.

LCM was found to be relatively well tolerated, with an adverse event rate of 41.9% at the 12-months. This was consistent with the incidence of adverse events that were reported in observational studies by Del Bianco et al., [14] and Casciato et al., [21] as well as the phase III, long-term, open-label study by Ben-Menachem et al., [22] (45.9%, 50.0% and 57.5%, respectively). The most common adverse reactions consisted of dizziness, somnolence, nausea/vomiting, depression, and double vision. We did not observe any unknown adverse events that were not reported in previous studies. Additionally, most adverse events were considered mild. Hence, we did not significantly change the treatment regimen for children and adolescents with LCM.

The strengths of this study are the longer follow-up. The major limitation of this study is investigators must rely on clinical records, as well as the fact that all children and adolescents with refractory epilepsy were treated by the same physician, it could be a limitation since the cases were not discussed with others. Nonetheless, this study may provide some real-world evidence for the effectiveness and safety of long-term adjunctive use of LCM for the treatment of refractory epilepsy within the children and adolescents population. LCM was found to be well tolerated, with relatively few adverse events.

In conclusion, this real-world study of 105 children and adolescents validated that LCM was both an effective and well-tolerated treatment option for the treatment of refractory epilepsy, even at long-term follow-up and low doses. These important findings suggest that LCM is likely to become a widely-used ASM for the treatment of epilepsy in clinical practice, and in children and adolescents.

## Data Availability

The data generated and/or analyzed during the present study are not publicly available due to the inclusion of private health information of patients at our institution, but may be available from the corresponding author upon reasonable request.
